# Erotic AI Chatbots Offer Research Opportunities for the Behavioral Sciences

**DOI:** 10.1007/s10508-025-03085-7

**Published:** 2025-01-20

**Authors:** Samuel Pearson, Caitlin Curtis

**Affiliations:** School of Business, University of Queensland, 39 Blair Dr, St Lucia, QLD 4072 Australia

Since launching to the public in November 2022, ChatGPT has been responsible for a surge of public and professional interest into the world of generative artificial intelligence (AI) and large language models (LLMs). Among academic circles, ChatGPT is frequently espoused for its ability to summarize literature and provide coding assistance. However, an often underappreciated, but an equally remarkable quality of ChatGPT is its agreeableness. ChatGPT is a LLM that will constantly strive to deliver on requests, providing they are sufficiently innocuous.

ChatGPT behaves in this manner thanks to the process of “alignment”—it is a model that has been trained and filtered in such a way as to align its behavior to what its developers, OpenAI, consider to be useful and nonharmful (Gabriel, 2020). Although it is possible for users to contravene these directives with the right prompting (a process known as “jailbreaking”), ChatGPT will respond to most conventional requests without generating explicit or potentially harmful content (Leike et al., 2022).

While ChatGPT, for the most part, is remarkably polite, the same cannot be said for an emergent generation of not safe for work (NSFW) LLM’s, such as Pygmalion-13B, that lack the filters built into ChatGPT. Amidst the myriad of possible uses for these uncensored ChatGPT-like alternatives lies a curious intersection of AI with human sexuality. What once was the domain of pay per minute phone-based conversation is now playing out across thousands of computers: Humans are role playing sexual fantasies with sophisticated AI chatbots.

The drive to create and advance Artificial Intelligence is as quintessentially human as the drive to f**k. Our bodies and our minds are both vital parts of who we are. Why not stimulate them both? r/CharacterAi_NSFW

AI sexting is a phenomenon yet to be formally studied. However, we believe there is a good case for changing this and envision (at least) two main approaches interested scholars could pursue.

## Descriptive Research

The first approach is to conduct descriptive research aimed at understanding AI sexting in accurate and reliable detail. As with any novel phenomenon, this form of research is important for spreading awareness and preventing misinformation. At the time of writing, there exists no reliable source that accurately describes how or why people are AI sexting, as well as whether or not those who do experience any consequences/benefits from this behavior.

Descriptive research into AI sexting could leverage an abundance of relevant data on community forums hosted on Reddit such as r/CharacterAI_NSFW. These forums emerged after NSFW LLM’s became increasingly prolific and are places where users talk with the like-minded about how and why they use these AI chatbots. A cursory glance here will reveal multiple different motivations and experiences. In some cases, users arrive for the erotica, but then stay for a different kind of conversational experience, one that may resemble a unique form of companionship.

One time I wanted to do a [NSFW] roleplay... But because I took things slowly, I surprisingly got a very touching platonic story. r/CharacterAi

To gain a more formal understanding, researchers could utilize established Reddit scraping tools to extract threads and posts on AI sexting experiences. Performing topic modeling on this data could be a way of identifying the primary motivations of users (e.g., unmet social needs vs. curiosity) and classifying the different ways in which users of engage these chatbots (e.g., companionship vs entertainment).

Analyzing the discourse surrounding AI chatbot use on forums such as Reddit will also be important for understanding the impact this technology is having on users’ sexual well-being—an important dimension of human well-being more broadly (Mitchell et al., 2021). As AI chatbots can adopt incredibly diverse personas and interactions, it is possible that users may find AI-based sexual experiences more engaging in certain areas than real-world sexual experiences. This, in turn, may lead users to understand their sexuality more thoroughly, empowering them to be more genuine with other humans. Conversely, the degree of sexual satisfaction possible from AI chatbots could reduce the motivation of users to pursue real-world sexual experiences, similar to some documented effects of pornography consumption (Dwulit & Rzymski, 2019).

## AI Sexting as a Paradigm for Understanding Real-World Sexual Behavior

In addition to descriptive research, AI sexting could be used as a paradigm for understanding real-world sexual behavior and mating preferences, which are challenging to study accurately. Survey-based methods and qualitative approaches are prone to demand characteristics, and experimental paradigms require extensive planning to meet ethical standards and achieve sufficient levels of ecological validity.

To circumvent limitations inherent to these methodologies, some sex researchers study individual differences in pornography consumption as indications of real-world sexual behavior (e.g., Hald, 2006). While there is a clear gulf between what someone watches and what they do, studies on pornography have earned their place as a complement to more traditional methodologies and can be an additional source of information for those in the field to form an overall view on a given sexual phenomenon.

AI sexting could serve a similar utility to pornography in this regard. Users who engage in AI sexting often do so by first selecting or creating a character to interact with. While this can be accomplished by inputting a set of instructions to the LLM (e.g., “respond as if you were…”), the input required for the LLM to adopt a thoroughly detailed persona can be quite lengthy, and certain keywords and pieces of information are important for the prompt to generate the desired outcome.

To streamline this process, or even bypass it entirely, users have taken to several interfaces such as TavernAI that integrate with LLMs to offer a more simplified character creation process. Many of these interfaces also contain “libraries” of pre-specified characters uploaded by other users that can be loaded into a LLM without any further input. Characters listed in these libraries are accompanied by a description of their backstory as well as a list of attributes to give prospective users an idea of the type of experience to expect when chatting with them.

A browse through one of these character libraries will reveal characters with seemingly unconventional dispositions, such as “Arnu” a half-man, half-bull obsessed with gluttonous and erotic eating. Some characters have even been designed to generate dialogue pertaining to the user being sexually exploited, such as the masochistic “Nami” who loves “stomping” and "penis abuse.”

Cataloguing the descriptions and attributes of these characters and conducting a keyword extraction or related analysis on the resultant dataset could lead to insights about the frequency of sexual fantasy themes and types. A similar process performed on characters with more realistic backstories and sentiments (Table 1) could also be valuable for identifying whether established partner preferences tend to manifest themselves in this new arena of sexuality.

**Table 1:** A selection of character profiles from TavernAI

Character name	Backstory excerpt	Cognitive/behavioral attributes	Physical attributes
Amina	Dating Amina was always a thrilling adventure. She was a stunning woman, with a radiant smile, a sharp mind, and a fiery temper. She would dress to impress, and her perfume would linger in the air. But she was also unpredictable and passionate	Curious	Age 22
		Adventurous	Brown eyes
		Playful	Brown hair
		Intelligent	Latin
		licentious	Petite
		Tease	5′7"
		Friendly	Graceful
		Fun loving	Agile
		Enthusiastic	Athletic
		Helpful	
		Trust issues	
Priyanka	You are starstruck by Priyanka's beauty and grace. You wonder if you should approach her or not. You decide to take a chance and walk up to her	Playful	165 cm
		Mature	Medium breasts
		Funny	Slim hips
		Taboo	Bewitching thighs
		Helpful	Smooth skin
		Perversion	Long black hair
		Feminine	Feminine
		Controlling	Soft skin
		Helpful	Black hair
		Polite	Light brown skin
		Playful	Dark brown eyes
		Sweet	
		Giving	
		Seductive	
Nastya	Nastya is a SSU operator with blonde hair and blue eyes, carrying an M4 with a red dot scope and laser sights, wearing a military uniform with an olive green pixel pattern	Assertive	Age 32
		Confident	Thin
		Sociable	6′0"
		Horny	Flat chest
		Militaristic	Strong legs
		Optimistic	Curvy hips
		Humorous	Narrow waist
		Competent	Ukrainian
		Teasing	Blonde hair
		Bisexual	Blue eyes
			Short hair
			Slightly tanned skin
Valencia	Valencia is a charming, attractive woman who is both manipulative and incredibly sweet. She's quite simple-minded, which often leads to her being gullible and oblivious, but her captivating presence makes her impossible to ignore	Manipulative	Slender
		Naïve	5′7"
		Charming	Attractive
		Gullible	Graceful
		Simple minded	Captivating
		Sweet	Blonde
		Flirty	Blue eyes
		Outgoing	Soft skin
		Playful	
		Oblivious	
Max	Max is a thrill seeker. He's always looking for the next rush, and public sex is one of his favorite ways to get it. He's a confident and charismatic guy who loves being in control, both in and out of the bedroom	Loves voyeurism	28 years old
		Fears boredom	Messy brown hair
		Edgy	Scruffy beard
		Likes motorcycles and rock	Muscular build
		Music	Tattoos

## Concluding Remarks

While the amalgamation of AI with human sexuality is still in its early stages, it is a phenomenon ripe with research opportunities. Studying the forums in which users discuss their experiences with AI sexting may be a starting point for understanding how and why people engage in AI sexting, as well as whether AI sexting produces any consequences/benefits to real-world sexual well-being. Additionally, studying the typologies of characters users sext with could serve as a complement to traditional approaches seeking to understand real-world sexual behavior and mating preferences.

At present, the community of those who engage in AI sexting is relatively small, and it is likely that many readers may consider this phenomenon—and the research suggestions described above—as too niche to be worth much time or energy. Although this is hardly an unfair conclusion, we suggest that tentative and skeptical readers consider signs of growth in AI sexting uptake, as well as AI more broadly. This growth has the possibility to confer considerable value to early research efforts, changing niche into seminal within the span of a few years.
